# Improving Primary Care Medication Processes by Using Shared Electronic Medication Plans in Switzerland: Lessons Learned From a Participatory Action Research Study

**DOI:** 10.2196/22319

**Published:** 2021-01-07

**Authors:** Benjamin Bugnon, Antoine Geissbuhler, Thomas Bischoff, Pascal Bonnabry, Christian von Plessen

**Affiliations:** 1 Institute of Pharmaceutical Sciences of Western Switzerland University of Geneva Geneva Switzerland; 2 Direction Générale de la Santé État de Vaud Lausanne Switzerland; 3 Department of Radiology and Medical Informatics University of Geneva Geneva Switzerland; 4 Department of Clinical Research University of Southern Denmark Odense Denmark; 5 Center for Primary Care and Public Health Unisanté Lausanne Switzerland

**Keywords:** shared electronic medication plan, medication list, medication reconciliation, electronic health records, primary care, national eHealth strategy, Switzerland, participatory action research, complex adaptive system, eHealth, medication, health information technology, implementation

## Abstract

**Background:**

Several countries have launched health information technology (HIT) systems for shared electronic medication plans. These systems enable patients and health care professionals to use and manage a common list of current medications across sectors and settings. Shared electronic medication plans have great potential to improve medication management and patient safety, but their integration into complex medication-related processes has proven difficult, and there is little scientific evidence to guide their implementation.

**Objective:**

The objective of this paper is to summarize lessons learned from primary care professionals involved in a pioneering pilot project in Switzerland for the systemwide implementation of shared electronic medication plans. We collected experiences, assessed the influences of the local context, and analyzed underlying mechanisms influencing the implementation.

**Methods:**

In this formative action research study, we followed 5 clusters of health care professionals during 6 months. The clusters represented rural and urban primary care settings. A total of 18 health care professionals (primary care physicians, pharmacists, and nurses) used the pilot version of a shared electronic medication plan on a secure web platform, the precursor of Switzerland’s electronic patient record infrastructure. We undertook 3 group interviews with each of the 5 clusters, analyzed the content longitudinally and across clusters, and summarized it into lessons learned.

**Results:**

Participants considered medication plan management, digitalized or not, a core element of good clinical practice. Requirements for the successful implementation of a shared electronic medication plan were the integration into and simplification of clinical routines. Participants underlined the importance of an enabling setting with designated reference professionals and regular high-quality interactions with patients. Such a setting should foster trusting relationships and nurture a culture of safety and data privacy. For participants, the HIT was a necessary but insufficient building block toward better interprofessional communication, especially in transitions. Despite oral and written information, the availability of shared electronic medication plans did not generate spontaneous demand from patients or foster more engagement in their medication management. The variable settings illustrated the diversity of medication management and the need for local adaptations.

**Conclusions:**

The results of our study present a unique and comprehensive description of the sociotechnical challenges of implementing shared electronic medication plans in primary care. The shared ownership among multiple stakeholders is a core challenge for implementers. No single stakeholder can build and maintain a safe, usable HIT system with up-to-date medication information. Buy-in from all involved health care professionals is necessary for consistent medication reconciliation along the entire care pathway. Implementers must balance the need to change clinical processes to achieve improvements with the need to integrate the shared electronic medication plan into existing routines to facilitate adoption. The lack of patient involvement warrants further study.

## Introduction

Medication processes are crucial for improving patient outcomes, and at the same time, medication-related errors are one of the main causes of the overall burden associated with adverse events [1]. Only 4% to 21% of patients receive the optimum benefits from their medication use [2]. Avoidable adverse drug events account for approximately 5% of hospital admissions [3]. According to the World Health Organization, a more responsible use of medicines could save up to US $42 billion annually worldwide by reducing medication-related harm [4,5].

Medication errors occur in all health care settings, but more commonly in ambulatory care [6] and during care transitions because of the loss or incomplete transfer of information about patients’ medications [7-9]. About 55% of patients risk having one or more unexplained differences in their documented treatment plans across different health care services [10]. The problem is ubiquitous and has an impact on patients and health care systems globally [7], including in Switzerland [11-13].

Medication reconciliation (MedRec), the process of creating and managing the most accurate list of the medications that a patient is taking [14], can prevent such events at interfaces of care. However, it is difficult because medication regimens are increasingly complex [15] and multiple disparate actors are involved [16,17]. It is perhaps unsurprising that despite significant efforts to implement it, MedRec is still only progressing slowly in many countries [17]. For example, in Switzerland, systematic MedRec has only been tested in a few pilot projects and has yet to be implemented across the whole country [11].

Health care organizations have invested in health information technology (HIT) systems to address these difficulties [18]. Such systems should help overcome insufficient access to up-to-date information, low efficiency, and organizational issues [17]. Moreover, they should help reduce stress among patients and workloads among staff caused by lack of information while avoiding risky workarounds and improving the quality of care [19]. Although the great potential for HIT investment is acknowledged internationally, approaches and strategies vary [20].

Several countries have launched HIT systems for shared electronic medication plans, which allow multiple health care professionals to use and manage their common patient’s current list of medications [20-22]. The core information in a shared electronic medication plan system is made up of the clinical decisions related to the treatment plan, such as adding, adapting, or stopping medications. The architecture used for a shared electronic medication plan system (eg, in Denmark [23]) contrasts with that of other systems that automatically calculate a patient’s current medication list from dispensing and e-prescribing databases (eg, France [24], Ireland [25], Netherlands [26]), but not all clinical or self-care decisions necessarily end up on paper, in an electronic prescription, or in dispensing notes. The latter automatic systems, therefore, appear limited in terms of information accuracy, whereas a digital shared medication plan fundamentally relies on the system’s joint and regular use in clinical practice to ensure consistently reconciled medication information along the patient’s entire care pathway.

Implementing HIT systems for shared medication plans is challenging. System usability and its integration into clinical workflows is essential for medication list accuracy [23,27,28]. Attention should be paid to clinical and administrative workflows and system design [22,29,30] as well as to easily accessible information technology and clinical support [31,32]. The need to clarify professionals’ responsibilities has often been raised [28,33,34]. Similarly, introducing a predefined process for using and managing patients’ shared medication plans has been claimed as a solution [30,31,33]. In addition, trust must be built into the system by making the shared information reliable [27,28,34] and ensuring the privacy and security of data [28,32,35]. Unfortunately, evidence-based strategies for implementing such a system cannot be derived from these often heterogeneous and highly contextual studies.

However, these studies have illustrated the sociotechnical nature and complexity of implementing a digital shared medication plan. Systemwide HIT implementation projects should embrace this complexity and consider strategic, managerial, and social aspects in addition to technological challenges [36-38]. One approach is using formative research to create collaborative learning opportunities in these complex situations [39-41]. Such research aims to interpret and understand the potential effects of an HIT implementation project rather than predict them. Insights into the key mechanisms affecting the success or failure of complex programs of change, such as the implementation of shared electronic medication plans, can support stakeholders as they seek to build on local experiences.

With this in mind, we designed a formative action research study of a pioneering Swiss pilot project using shared electronic medication plans on an eHealth platform. We aimed to produce practical knowledge for use in the implementation of shared electronic medication plans on a larger scale. The study objectives were to learn from the local experience of 5 clusters of primary care professionals, assess the influences of context, and describe related mechanisms in order to achieve the efficient use of digital shared medication plans for safer, more effective patient care.

## Methods

### Design

This formative participatory action research (PAR) study followed 5 clusters of health care professionals over 6 months using 3 interviews per cluster and a model to guide an iterative inductive thematic analysis.

PAR is a collective, self-reflective investigation undertaken by researchers and participants together [42]. It connects actions influenced by context, culture, and history and is embedded in social dynamics. The strengths of PAR are responsiveness to context, the engagement of frontline health care professionals, and a focus on the mechanisms of implementation that can help bring about real-world service improvements [43].

Throughout the successive meetings with participants, we followed the iterative process proposed by Loewenson et al [44], using the steps of systematizing experience, collectively analyzing and problematizing, reflecting on and choosing an action, taking and evaluating action, and systematizing learning. We invited each group of participants to define their collective commitments at the first meeting. The reflective process was stimulated by asking questions such as “What is going on?” “How do we continue?” and “What are our main lessons learned?” We also ensured that all the lessons learned that were documented by researchers were proposed for further discussion or refinement.

### Context

**Figure 1:**
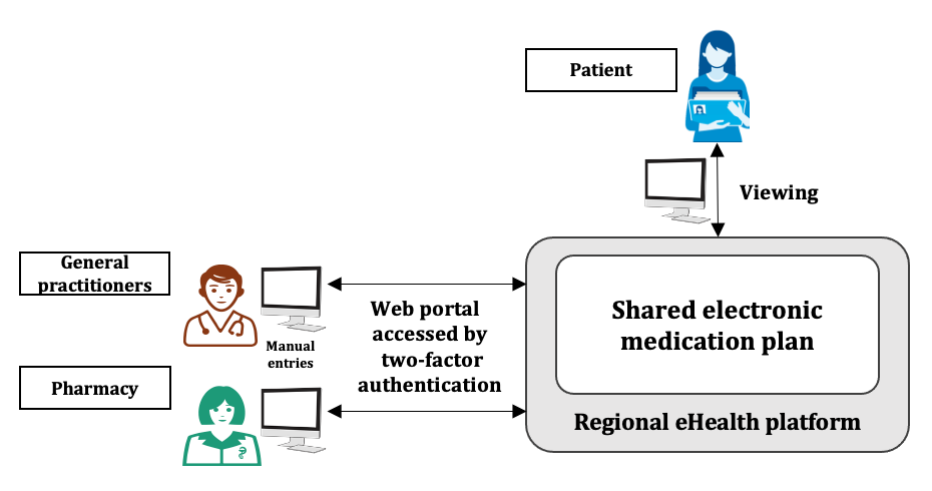
Description of the shared electronic medication plan system used in the regional pilot project. The web portal was not integrated with usual systems used by care professionals, as the national standard for e-medication based on IHE Pharmacy HL7 CDA was a work in progress during the pilot.

The study was embedded in the pilot project for the implementation of shared electronic medication plans on the regional eHealth platform for the Nord Broye region in the canton of Vaud [45]. We recruited health care professionals into local study clusters from among the 36 general practitioners (GPs) and 36 pharmacies who had cared for the 193 patients participating in the pilot project from 2013 to 2018 (Figure 1). Primary care professionals were free to enroll in the pilot project led by the regional network for care coordination, which was sponsored by their respective corporation and public authority. Patients using at least three medications regularly were invited to join the pilot project’s medication management program. Care professionals communicated to patients directly, while the pilot project team provided leaflets and information online. They nominated a GP and a pharmacy as reference points to manage their medications, and they committed to consulting and procuring their medication only from them while sharing all necessary information completely.

The digital solution chosen for the shared medication plans was an online platform for creating, using, and managing a list of all the medications a patient was taking and had taken in the past [22,46]. A shared electronic medication plan must be accessible, complete, and updated at every contact between the patient and an intervening health care professional. Technologically, this solution was envisioned as an interoperable system based on the pharmacy profiles defined by the Integrating the Healthcare Enterprise (IHE) consortium [47]. During the pilot project, the definition of a national e-medication interoperability standard based on IHE pharmacy HL7 CDA was under development (interprofessional working group from 2015, recommendation published in 2017) [22,48]. The users accessed the shared medication plan through a secure web portal with two-factor authentication. Patients could access their medication plan online or receive a printed one. Professionals had to enter all data manually in addition to filling out the usual paper documentation because their clinical software applications were not yet integrated.

The solution is a module of the web platform developed for the cantons of Geneva and Vaud in anticipation of Switzerland’s electronic patient record (EPR) system, a national digital inventory of all the relevant health data concerning the country’s patients [22]. The EPR is based on decentralized information exchange infrastructure. Several regional platforms have been implemented that are run by private or public entities but overseen nationally by the federal law of 2017 [49]. Patients own their data and share them with health care professionals of their own free will. Primary care physicians are free to choose whether they want to join the EPR (opt in), whereas all hospitals are obliged to be connected. Swiss national policy acknowledges the importance of e-medication [50,51], but an overall strategy has not yet been defined.

Switzerland’s political culture is liberal, and the 26 cantons of the federal state have far-reaching autonomy regarding the organization of health care [52]. Patients can access the health care professionals or specialist physicians of their choice. GPs only have a gatekeeper function in some optional insurance plans. As of 2020, there are no shared patient registers. Among the member states of the Organisation for Economic Co-operation and Development (OECD), Switzerland has below-average digital maturity [53], and 30% of its GPs still use paper-based patient records, far behind their colleagues from the European Union, of whom only 4% rely on paper. Finally, there are no regulatory or other specific incentives for the vendors of medical or pharmaceutical record software.

### Theoretical and Conceptual Framework

Organizational innovations in health care often fail because the complexity and adaptability of the health system are underestimated [54-56]. Recognizing health care as a complex adaptive system (CAS) means focusing on the dynamic interactions between individuals and organizations across the entire system. When seeking to initiate change in a CAS, sensemaking and learning about it are more important than planning and controlling the change itself. By definition, a CAS is unpredictable, but some simple rules (ie, guiding principles), can help foster transformation [55,57-59].

**Figure 2:**
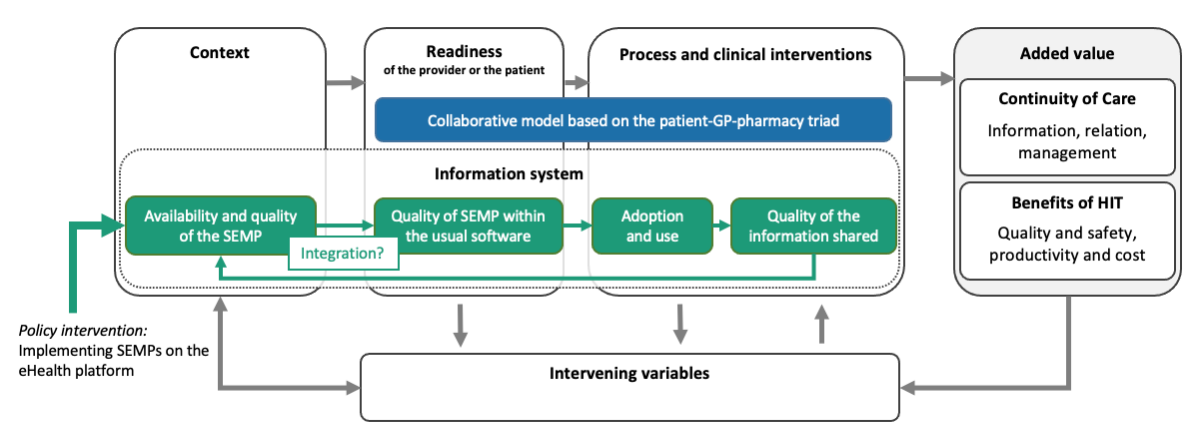
Proposed model for the implementation of a shared electronic medication plan system. GP: general practitioner; HIT: health information technology; SEMP: shared electronic medication plan.

Creating a common electronic patient health record incorporating a shared medication plan is in itself a complex sociotechnical intervention; the technological component of the intervention is influenced by and influences every user’s behavior, as well as the organization and context [60]. We developed a model (Figure 2) of how different elements of the implementation of the shared electronic medication plan might be linked to expected results.

We based our model on Lilford et al’s [61] approach to mapping policy and service interventions with regard to structures, processes, outcomes, and intervening variables*.* In the pilot study, the introduction of the shared electronic medication plan system affected the participating health care organization structures and required adaptions of their work processes. Some participants also combined the shared medication plan use with other clinical interventions, such as medication review. All these elements as a whole system led to health care outcomes. Although we did not want to predefine the intended outcomes of safer and better patient care, we did specify that the continuity of care, claimed as a main policy ambition for the pilot project, should be not only at the informational level but also at the relational and management levels, as per Haggerty et al’s [62] definition. It is also essential to consider the intervening variables, as they are interrelated with the structural and process factors mediating the outcomes. For instance, a patient’s trust in their pharmacy and its staff (intervening variable) is influenced by the availability of a space in the pharmacy where they can talk in confidence (structure), whether a dedicated pharmacist follows up with a chronic patient (generic process), how information is given when dispensing a pillbox (clinical process), and the consequent safe use of medicines (outcomes). In the present study, the main intervention is at the policy level: implementing shared electronic medication plans on the eHealth platform in the region. Our study sought to leverage health care professionals’ experiences to assess contextual influences from a systemic perspective. For this reason, the model specifies both the context and the readiness of the provider or the patient, as structural factors can be respectively external or internal of the health care providers.

The information system itself was added to the model as a transversal dimension, based on the eHealth Clinical Adoption framework defined by Lau et al [63]. Those authors described how the successful adoption and benefits of HIT depend on its quality. The overall quality of HIT is made up of the qualities of the system, the service, and the information available. For a shared record system, because the quality of information is made up of shared content, it is strongly dependent on the quality of usage. This is why our model illustrates the interrelation of the perceived quality of the shared electronic medication plan system, the quality of its usage, and the quality of the shared content as distinct dimensions. Finally, the model describes the shared electronic medication plan’s overall added value in terms of the improved elements in the continuity of care and the benefits of HIT.

All the dimensions in our model helped us to break down and make sense of the implementation of the HIT, potential interventions, and the points requiring study. The two essential new elements brought in by the pilot project were the addition of a shared electronic medication plan system onto an existing eHealth platform and a new collaborative model based on the patient-GP-pharmacy triad to make primary care medication management safer in cases involving polypharmacy.

### Recruitment in the Study, Sampling, and Ethics

Invitations were sent to the group of 36 GPs and 36 pharmacies who had been enrolled in the pilot project. From those who volunteered, we created clusters consisting of at least one pharmacist from an enrolled pharmacy and one GP who were responsible for at least one common patient enrolled in the pilot project. Each cluster could invite other primary health care professionals involved in their local settings, such as a home care nurse. We characterized each cluster by urbanization density classification [64]. All participants consented according to the canton of Vaud’s legal, privacy, and ethical requirements. No patient data were collected.

### Data Collection and Analysis

**Figure 3:**
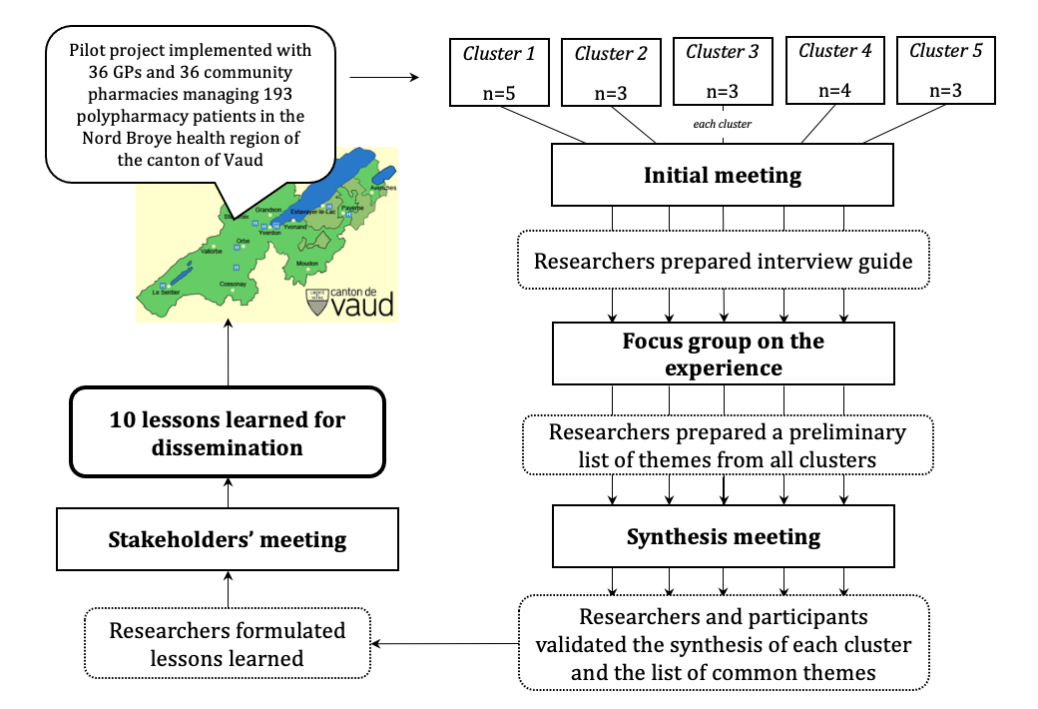
Data collection and analysis. GP: general practitioner.

Two researchers, a pharmacist and GP respectively, both with research training and experience, collected and analyzed the data (Figure 3). They were not enrolled in the pilot project (ie, did not count in clusters) but were familiar with the settings, and they knew some of the participants professionally.

Between May 2018 and January 2019, each cluster participated in 3 group interviews whose main topics of investigation were, respectively, (1) motivations and commitment, (2) experience and refinement, and (3) synthesis and learnings. Interview guides (Multimedia Appendix 1) for each round were prepared using the conceptual model as a basis. The investigators guided participants toward thinking about the added value of the eHealth platform and the collaborative model of care as 2 interdependent components associated with the implementation of patients’ shared medication plans. Participants were first encouraged to share and reflect on their experiences of initiating and managing the medication plan, using the platform, interacting with patients about medication lists, and collaborating with other professionals. These experiences then nurtured discussions on the contextual or organizational factors influencing implementation and on the role of shared electronic medication plans in achieving safer, more effective care. The investigators facilitated exploration of the different themes that emerged from each group in order to increase diversity across clusters. During the last cluster meeting, participants also discussed and summarized the most important practical knowledge that should be disseminated to stakeholders involved in the future development and scale-up of shared electronic medication plan systems.

Data collection and analysis were iterative so that each group’s experiences could be collected and synthesized longitudinally and data across clusters could be analyzed horizontally to condense them into themes. Finally, we conducted a secondary thematic content analysis to condense the themes into lessons learned, or lessons intended to describe the simple rules underlying the mechanisms related to the implementation in a CAS.

Following the principles of PAR, we proposed refinements to and requested validation from participants at each step of the study. Furthermore, we presented the lessons learned at a stakeholders meeting, which included participants and representatives of other stakeholders in the regional pilot project. This gave time for discussions and dialogue on setting prioritized next steps. We also attempted to enhance the reliability of our research by having data analyzed by the 2 main researchers and then by a researcher outside the pilot project [65].

## Results

### Participants

Among the 36 GPs and 36 pharmacies who were enrolled in the pilot project, 31 volunteered for this action research study. A total of 5 clusters were identified, including 13 care professionals. Consequently, 18 volunteers had no patients in common with any other volunteer professionals and thus were excluded in this study. The 13 participants invited 5 extra primary health care professionals into their clusters, for a total of 18 participants (Table 1). We conducted 15 group interviews that lasted 60 to 105 minutes.

**Table 1:** Characteristics of the 5 clusters.

Cluster	Cluster location	Participants
1	Town (semidense)	GP^a^, 2 pharmacists from different pharmacies, a medical secretary specialized in care coordination, and a home care nurse
2	Town (semidense)	GP also working in local hospital emergency unit and 2 pharmacists from different pharmacies
3	Rural area (dispersed)	GP, pharmacist, and independent nurse in GP practice
4	University center for primary care in a city	GP, 2 pharmacists, and scientific collaborator
5	City	GP, pharmacist, and home care nurse, all responsible for a nursing home

^a^GP: general practitioner.

### Lessons Learned

An overview of the lessons learned is presented in Textbox 1.

**Lessons learned.**

**Lessons learned, to be used in the strategy for the systemwide implementation of shared electronic medication plans improving primary care medication processes**

1. Information sharing during clinical routines must be simplified and secured by integrating shared electronic medication plans into existing processes and health information technology systems.
2. A medication plan, whether digital or not, is a matter of good clinical practice.
3. Designating reference professionals ensures the exhaustivity and continuity of the medication information communicated.
4. Regular high-quality interactions between patients and professionals strengthen the continuity of medication plan management.
5. Implementing a new tool, ensuring good clinical practice, and increasing interactions for coordination require more resources and an adapted organizational model.
6. The availability of the shared electronic medication plan did not generate spontaneous demand from patients or foster more engagement in their medication management.
7. Adopting a shared electronic medication plan is triggered by a culture of patient safety and data privacy.
8. Fostering trusting relationships at all levels is essential.
9. Legal, financial, and governance framework conditions influence the uptake and impact of shared electronic medication plans.
10. A shared electronic medication plan is a necessary building block of communication about medication, especially at transitions, but it is not a sufficient one.

#### Lesson No. 1: Information Sharing During Clinical Routines Must Be Simplified and Secured by Integrating Shared Electronic Medication Plans Into Existing Processes and HIT Systems

Participants consistently emphasized the need to integrate the shared electronic medication plan system into their usual electronic medical records systems and pharmacy management systems:

Its integration into my usual software is crucial to simplifying my work.

GP, cluster 3

The workflow is sometimes intense… and we are a team… only integration can enable reliable information sharing on any contact with the patient.

Pharmacist, cluster 5

During the pilot project, participants had to document the medication-related decisions in both the shared system and their usual patient record system. They feared this double documentation could cause errors, and they expressed frustration about redundant work:

For the small number of patients we are following [about 10], it’s okay, but we couldn’t do it properly for every patient without a certain degree of automatization and integration with our usual system.

Pharmacist 1, cluster 2

The shared electronic medication plan system’s overall good usability and integration with current clinical software was considered a sine qua non for meaningful implementation.

Participants highlighted integration issues as crucial, and they deplored their dependence on their software vendors to better integrate the shared electronic medication plan in their own system. They were critical of the national strategy, which foresees standards of interoperability but leaves system integration to market forces:

As clients, we are captives of our medical software vendor. What can you [the public administration] do to leverage integration?

GP, cluster 3

Poor current levels of competition in the market for medical records or pharmacy systems was also mentioned as a barrier to integration.

#### Lesson No. 2: A Medication Plan, Whether Digital or Not, Is a Matter of Good Clinical Practice

Participants proposed that professional attitudes and clinical work processes were even more important than HIT systems for improving medication management:

You [the investigators] are working to set up a great, relevant system…but we could likely do better with a less sophisticated tool.…Working with a medication plan should be a matter of good practice!

GP, cluster 1

During group discussions, participants mentioned that prescribing drugs without a holistic view of all the medications a patient is taking and communication of the current medication plan to other health care professionals involved were both not uncommon.

Notwithstanding that health care professionals are legally responsible for the safe use of medications, the responsibilities for creating, maintaining, and communicating medication plans were not always clear. Multiple physicians write prescriptions, but they do not always maintain an overview of the patient’s entire list of medications, which risks causing the patient serious problems. For example, participants revealed that some older patients accumulated numerous medications from various prescribers with no awareness of the potential for drug-drug interactions. It was argued that procedures, standards, or even regulatory actions were needed to clarify responsibilities, regardless of the implementation of any new HIT systems.

#### Lesson No. 3: Designating Reference Professionals Ensures the Exhaustivity and Continuity of the Medication Information Communicated

Participants recognized that formalized roles and relationships between patients and their GPs and pharmacists improved the exchange of information about medications. During the pilot project, patients registered with one GP and one pharmacy as health care professional reference points and committed to sharing all their medication-related information with them. This was a major change from the usual practice in Swiss health care:

When she enrolled, one of our patients informed us that she received a neuroleptic drug from a specialist by post. None of us knew! She is a typical polypharmacy patient who regularly comes to us for her medicines.…We did not expect that from her at all.

Pharmacist, cluster 3

Formalizing these associations led to more accurate medication lists through improved relational continuity and clear channels of communication with other health care providers and professionals, such as hospitals or specialist physicians. These formal reference persons were also seen as key facilitators during the scaling-up transition period from multiple sources of medication information to one systematically used shared electronic medication plan system.

#### Lesson No. 4: Regular High-Quality Interactions Between Patients and Professionals Strengthen the Continuity of Medication Plan Management

Whereas the shared electronic medication plan improves documentation and information exchange, the validity and relevance of information about medications depend on the quality and regularity of the interactions between patients and professionals:

After some time…after doing regular reviews and interacting with the patient,…that’s how you get to know—when the trust is built—the things that matter to them, their worries…and they may even confess how they really manage their medication! From there, you can really care for them and support them on their pathway.

Nurse, cluster 3

To illustrate this point, participants mentioned common activities, such as medication reviews, the identification of side effects, and the evaluation of adherence or support for administration. These interventions could also serve as important checkpoints for the accuracy of the medication plan. Furthermore, participants suggested that associating the implementation of shared electronic medication plans with these other important activities could accelerate their adoption.

#### Lesson No. 5: Implementing a New Tool, Ensuring Good Clinical Practice, and Increasing Interactions for Coordination Require More Resources and an Adapted Organizational Model

Not all primary health care professionals are equally ready to adapt their daily clinical practice for better patient follow-up and coordination activities. Although a shared electronic medication plan system has the potential to increase efficiency, the adoption capacity of providers depends on the availability of competent staff, flexibility, adequate facilities, and an effective organizational model:

With the pharmacy team, we have participated in several pilot projects on new services.…We hired an extra pharmacist…but the ones [ie, other pharmacies and their staff] that do not invest will likely not manage to evolve and will struggle more with the regular follow-up of patients who do have a [shared electronic medication plan].…

Pharmacist, cluster 5

We have now agreed on how we proceed with patients who are followed by the practice [from the cluster] and come to the pharmacy after hospital discharge…and that the nurse provides communication if there is a change.

Pharmacist, cluster 3

They highlighted that the introduction of new roles and competencies, such as the medical secretary specialized in care coordination, the independent nurses in GP practices, or the clinical pharmacist for pharmaceutical care, was still at the early stage of development in the region and that the financing model was not yet well established.

#### Lesson No. 6: The Availability of the Shared Electronic Medication Plan Did Not Generate Spontaneous Demand From Patients or Foster More Engagement in Their Medication Management

Participants reported that very few patients showed interest in exploring or using the shared electronic medication plan. Oral and written information given out at project inclusion and through promotional flyers in the waiting areas of GPs’ practices, in pharmacies, or online had not seemed to make a difference. Some speculated about explanations for this apparent lack of interest:

Some young and some elderly [declined access to the web portal]. It did not seem to be a matter of age, even if there were some technological barriers in some cases.

Nurse, cluster 3

They accepted [participating in the project] because I stated that it would be good for them.

GP, cluster 2

Patients seemed to have a limited understanding of the processes of medication management and had difficulties viewing its potential in terms of improvements to quality and safety. Accordingly, the rationale for the shared electronic medication plan and how it functioned remained obscure to them:

When we came to this patient, with all these forms, to ask him if he’d sign to agree that his regular GP and pharmacy—who he’d known for a long time—could communicate about his medication…he was like, “How come? You do that usually, don’t you?” He was very surprised and kind of worried!

Pharmacist 2, cluster 4

Apparently, this patient had taken it for granted that reasonable communication processes existed between his GP and his pharmacy. He had not been aware of the regulatory and practical barriers to sharing health-related information.

Study participants further argued that the intention behind the design of the shared electronic medication plan system had not been to engage patients:

The medication plan could also be a tool for extra interventions with the patient, like patient education, but it can also just be simply printed from our software.…At the moment,…the [shared electronic medication plan] isn’t designed as a specific tool to foster patient engagement.

Pharmacist 1, cluster 4

Thus, to date, patients have not been considered active participants in their medication management, and the HIT system was not designed to foster patient empowerment.

#### Lesson No. 7: Adopting a Shared Electronic Medication Plan Is Triggered by a Culture of Patient Safety and Data Privacy

Participants noted the ambiguity between sharing health-related information to improve medication management and safety and the need for data privacy and confidentiality:

It’s a question of balancing benefits and risks. Chronic patients with polypharmacy are more likely to benefit and realize its importance.

GP, cluster 5

The fear of privacy related to digital technology, often fueled in the media, can hinder adoption. Participants pointed to the need to address habits and culture during the shared electronic medication plan system’s implementation:

The use of shared records is essential for medication safety, but this challenges habits and perceived responsibilities, especially among older generations of doctors. This cultural shift should be supported, and it should start with new doctors during their education.

GP, cluster 4

Transparent evaluation was identified as a means of demonstrating the clinical benefits and nurturing a dialogue on privacy and patient safety.

#### Lesson No. 8: Fostering Trusting Relationships at All Levels Is Essential

Participants repeatedly highlighted the importance of trust in the implementation of the shared electronic medication plan:

It [the shared electronic medication plan’s use by the patient-GP-pharmacy triad] should be based on trust.

Pharmacist, cluster 1

Trust between patients and professionals is required for medication plans to have any value; trust between professionals fosters information exchange; and trust between HIT providers, health care professionals, and the state facilitates implementation. Trust in the HIT can be diminished by breaches of confidentiality and the misguided implementation of eHealth systems. Conversely, participants appreciated the present study’s collaborative design because it fostered trusting relationships among them:

It [participating in the study] brought us around the table, gave us time to get to know each other and discuss.…Although we regularly interact, it is always brief.

Pharmacist, cluster 1

It [participating in the study] helped to reach a better mutual understanding and create a climate of collaboration.

GP, cluster 2

#### Lesson No. 9: Legal, Financial, and Governance Framework Conditions Influence the Uptake and Impact of Shared Electronic Medication Plans

Group discussions repeatedly mentioned the crucial importance of the legal, governance, and financial conditions surrounding medication management. Questions were raised about the mandatory or facultative use of the shared electronic medication plan system, its legal status, and different users’ legal responsibilities in the case of adverse events, discrepancies, and incompleteness:

If the [shared electronic medication plan’s] use were mandatory by law [for all health care professionals], at least then I’d think that we could rely on it more.…If not, you will always wonder if it is complete or not….You’re supposed to trust the list, not just consider if it’s the truth or not when you are making decisions.…

GP, cluster 2

In the pilot project launching phase in particular, concerns were raised that a lack of professional adherence would impede scale-up:

I need to be sure the plan is complete and updated.…If not, I won’t use it. But if everyone avoids using it for the same reason,…no one will ever update it.

GP, cluster 5

Indeed, the participants were divided about whether to make the shared electronic medication plan mandatory. Some emphasized the legitimacy of an official status, arguing for mandatory participation for all health care professionals. Others advocated for a more specific strategy to enhance the involvement of health care professionals, for example, via financial incentives for both patients and health care professionals when they signed up for a collaborative model of care.

Participants were concerned about the shared electronic medication plan system’s governance and how their active involvement to manage it would be financed. Here, they perceived the liberal approach to organizing Switzerland’s health care to be a major challenge:

It is important to clarify the roles and responsibilities….But who should decide?

Nurse, cluster 1

The current model of reimbursement for health care professionals’ activities was also considered a barrier because of its poor financial incentives for collaborative care management activities and the lack of consistency among reimbursement models:

Updating the plan, making sure it is complete; explaining; answering questions the patient may have—it all takes time! But to date, we are not directly paid for this.…The negotiations with the health assurance companies [ie, the payers in the system] are going to be complicated.

Pharmacist, cluster 3

One example of the inconsistency in health care professionals’ payments is the support for medication management:

Patients we have known for a long time…suddenly disappear because the GP calls for homecare services to follow-up. They prepare the pillbox at the dining table while chatting with the patient or the family—there is a much higher risk of errors than in a secure double-checked process in the pharmacy. We often know the patient’s preferences, their habits, history, story,…but we are not involved anymore.

Pharmacist 1, cluster 1

Even when there is a local consensus, the financial reality is that:

For homecare services, it’s the way they are financed for entering the home to better assess and follow-up a situation that is getting more complicated.…The psychosocial support is not really reimbursed.…”

Nurse, cluster 1

The current reimbursement system for coordinating activities (especially in complex cases), reviewing medication, and supporting patients with their medication use and adherence was perceived to be a hindrance to regular, in-depth updating of the shared electronic medication plan. Switzerland’s general governance and financing systems for health care services may themselves pose a challenge to safe and meaningful scale-up of shared electronic medication plans.

#### Lesson No. 10: A Shared Electronic Medication Plan System Is a Necessary Building Block of Communication About Medication, Especially at Transitions, but It Is Not a Sufficient One

While participants appreciated the shared electronic medication plan as a useful building block in a system for medication management, they cautioned that communication problems, especially during transitions, were much broader:

All the issues related to care transitions go beyond the scope of medication information….You need to take into account many factors to adapt care, starting from the patients’ pathways and their specific medical conditions.

GP, cluster 2

For example, participants mentioned that few hospital units had properly implemented MedRec and that the introduction of shared electronic medication plans alone would not directly change that.

Participants lamented the lack of standardized communication, especially between the GPs or the pharmacy and home care services or hospitals:

Actually,…communication between the pharmacy and the doctor works pretty well. We work with the prescriptions, and sometimes we call each other if needed,…but the main issues are with the multiple homecare services organizations operating for our patients.…Even public organizations work in different ways [eg, medication management, communication of lists].

GP, cluster 3

Most of the troubles come when the patient’s hospitalized….

GP, cluster 1

They highlighted the risk of losing or misunderstanding information due to heterogeneous communication habits and multiple channels of exchange. They hoped that the shared electronic medication plan system would contribute to the standardization of medication information and encourage better communication among professionals.

Overall, participants highlighted that the shared electronic medication plan system had the potential to trigger improvements beyond its original specific scope:

It’s like a big, complex ball of wool, with many knots….You have to start somewhere, to pinch one strand to start untangling it.…You cannot pull it in all directions at once.

GP, cluster 2

## Discussion

### Principal Findings

Health care professionals and patients alike need an accessible, common, complete, and accurate list of all the medications the patient is taking. However, introducing shared medication plans has proven difficult in several countries, and guidance for their implementation seems needed. We have presented 10 lessons learned from the first pilot project in Switzerland attempting to implement shared electronic medication plans, and we discuss this in light of studies from other contexts.

Clearly, no single organization can create and implement a comprehensive, robust, and user-friendly shared electronic medication plan system alone; HIT companies, policy makers, project teams, and the system’s users—both professionals and patients—must also collaborate. Given the systemic and safety implications of implementing eHealth projects, public health authorities are taking significant steps to improve the usability of HIT systems [66]. The pilot project suffered from a lack of cooperation among HIT, clinical, and policy stakeholders and from weak enabling framework conditions, especially at the federal level. These external issues prolonged the project phase, contributed to the lack of evolution of the eHealth platform and the absence of integration with other HIT applications, and ultimately led to disengagement by health care professionals. Usability “does not heal by itself” [67] through market competition. Federating the stakeholders in an appropriate, adaptable framework involving collaboration and policy coordination is a sine qua non for the successful implementation of ambitious eHealth projects. Building a shared electronic medication plan system implies a shared ownership.

Implementing a shared electronic medication plan system and improving clinical practice is a complex process. Stakeholders face a dilemma. On the one hand, better clinical practice requires change, which technology can support. On the other hand, the new technology needs to be fitted to an existing process to increase its acceptance. Study participants emphasized that improvements required good clinical practice, trust, and collaboration. Technology alone, therefore, is clearly neither a prerequisite nor a guarantee for safer work processes; rather, it acts as a catalyst [60] for the simultaneous innovation of the technology, processes, and relationships [68,69]. eHealth platforms could be better implemented by using approaches from quality improvement [70] and service design [71]. Combining HIT system design and clinical practice improvement within a shared electronic medication plan’s implementation strategy could likely prevent the chicken-or-the-egg dilemma and better leverage synergies.

A shared electronic medication plan should improve coordination in variable and changing contexts by using the same common regional HIT system. We accepted any proposition from the participants and found variability in how the shared electronic medication plan was initiated and updated across the 5 clusters of health care professionals. In the clusters, the main professional who regularly reviewed and updated the shared electronic medication plan was different; in 1 cluster it was a pharmacist, in 2 it was a GP, and in 2 it was a nurse or the medical secretary with a care coordination role in the GP practice. The basic rule was that they needed to define how they would manage the shared medication plan together in routine practice to ensure its accuracy. The model was easily adopted in every case because it was based on a consensus and the professionals’ preferences on how they wanted to manage it. Participants acknowledged their inherent shared ownership of the shared electronic medication plan. At the same time, clear processes and responsibilities are called for, both in HIT design and among health care professionals [28,32]. Our findings suggested that there was no one-size-fits-all solution; thus, strictly enforcing the implementation of a rigid solution could be difficult and could cause unintended consequences, and it would be unlikely to be achieved through policy making in Switzerland’s context. A strategy enabling all health care professionals to be involved in a patient’s care via a shared system and promoting basic principles of use seems more appropriate. Such an approach facilitates regular updates directly when interventions are made or discrepancies are identified. It may also increase the sense of shared ownership and favor self-organization at the local or the patient level. Knowing the issues related to the complex workflows that hinder the implementation of MedRec [17], an eHealth platform will not likely solve every problem. Standardization, automation, user constraints, and clear roles and processes [28-31] all need to be carefully balanced, with room for adaptations to local variables, in order to support a mutual commitment to using patients’ common medication plans along the continuum of care.

The process of managing a shared electronic medication plan also raises questions about patients’ roles and responsibilities. The pilot project HIT system implemented in the present study was not designed to empower patients and facilitate their engagement in their own medication management. The only function available to patients via the patient portal was a view of their medication plan. They could not make adjustments. The limitations of such an approach are obvious: safe, efficient medication management requires contributions from all stakeholders, including patients and their relatives. Despite increasing evidence of the benefits of comanaging digital medication systems with patients [72-75], they are still mostly treated as the passive recipients of medication lists produced by and for health care professionals. The German experience is insightful. Despite a clear policy for the systematic production of medication plans by professionals, expectations have fallen short. Few of the eligible patients ended up with the accurate list they were supposed to have [76], and when they did, only about half of them understood its content [77]. We plan to further study patients’ perspectives in our ensuing work. Today, any service improvements or innovation should acknowledge the coproduction of value by patients and health care professionals together as partners [78].

Guiding stakeholders’ actions towards the meaningful use of shared electronic medication plans should start by acknowledging the shared ownership and complexity of the process. From a CAS perspective, a strategy for driving major changes relies on the power of an attractor [79], a vision shared among stakeholders, that can inspire independent people and organizations to self-organize and evolve in a coherent, synergistic manner within the broader health care system. Advocating for a shared electronic medication plan comanaged by patients and health care professionals as a shared vision is even more important in settings where stakeholder fragmentation and autonomy are high. Our study stimulated collaborative actions by raising awareness of the value and shared ownership of a shared electronic medication plan, which encourages leadership at every level and supports collective learning. Indeed, these are some of the key ingredients for successfully enabling transformation in a CAS [57,80,81].

### Strengths and Limitations of Our Participatory Action Research Study

Mobilizing stakeholders through formative action research is a promising approach to dealing with the complex sociotechnical challenges related to shared electronic medication plans. This type of research can nurture the implementation dynamic; policymakers cannot mandate the required motivation and trust. Local networks and cultures can vary and have a significant influence on whether a new shared HIT system gains acceptance. Disregarding them has contributed to ineffective communication with the public or failure to engage with health care professionals [38,82]. Health care professionals want to be considered long-term partners in major HIT projects, not simply clients [83]. The series of cluster meetings during our study helped to enhance mutual understanding, collective learning, and trust. Similar benefits have been reported from facilitating an interprofessional dynamic [33,84], especially when it was a core focus of the implementation strategy [29,34]. Our study participants rarely have opportunities for dialogue and reflection at the local level, and this was appreciated and even triggered some further collaborations. We also realized that the mixed status of our 2 main investigators, who were clinicians, researchers, and employees of the public health authorities, strengthened our participants’ motivation to get involved in the study. They considered involvement to be a meaningful way to facilitate communication and mutual understanding between the people in the field and decision makers. We argue that further formative action research could be a key facilitator in the implementation of new shared electronic medication plan systems.

Our study has some limitations. First, we only included GPs, pharmacists, and nurses involved in primary care. Thus, we could only investigate issues related to care transitions from the primary care perspective. Second, we report experiences from a relatively small region of French-speaking Switzerland, which might limit the study’s transferability to other contexts. The lessons learned could, nevertheless, support learning in other settings. Third, the participants who volunteered for the study were likely early adopters and highly motivated. Additionally, because implementation intensity was low, some specific use and implementation issues that are likely to be encountered in the future require more assessment.

However, our novel approach, which used 5 clusters and iterative participatory analysis, is a strength of our study. We maximized diversity by including rural and urban settings, whereas earlier studies were mostly limited to university medical centers. Early adopters are not the majority, of course, but they are often the determinants for the diffusion of any innovation. Leveraging their experience can benefit other individuals less keen to explore that innovation. Moreover, we sought to embrace complexity by using a systems perspective to support sensemaking and awareness. These can help guide stakeholders and likely support further learnings.

### Conclusion

The 10 lessons learned from this study give an overview of the mechanisms and dimensions related to the implementation of shared electronic medication plans in primary care settings. This paper gives practical guidance on implementation and describes some of the key sociotechnical challenges that will face implementors aiming to instill the regular, meaningful use of shared electronic medication plans—plans that should be consistently reconciled along the patient’s entire care pathway—in clinical practice.

We consider the poor spontaneous patient involvement with their shared electronic medication plan to be a significant shortcoming and a point that has clearly not met the policy ambitions of fostering patient empowerment and medication adherence. Nevertheless, the local adaptability of the participating clusters was striking, as was their ability to reach consensus around useful solutions. This suggests that implementation strategies should facilitate the emergence of local engagement rather than implementing rigid top-down processes. HIT systems should be able to support various configurations of use in practice while maintaining predefined basic principles agreed among stakeholders. Last but not least, collective leadership is essential to handle the inherent shared ownership of a medication plan and to make change happen at every level, from direct patient care to the policy framework.

Future research should explore experiences in different countries in order to determine how system characteristics, stakeholder cooperation, health care policy, patients’ and professionals’ responsibilities, and implementation strategies affect the uptake of such shared systems by health care professionals and the benefits these shared medication plans bring to patients and health care services overall. Integrating patients so that they begin to comanage their medication plans also raises important questions. Finally, we suggest that formative participatory action research, including qualitative and quantitative methodologies, should play a key facilitating role in achieving a safe and meaningful use of shared electronic medication plans to create an efficient learning health system.

## Appendix

Multimedia Appendix 1 Interview guide and meetings goals.

## Abbreviations

CAS: complex adaptive system
EPR: electronic patient record
GP: general practitioner
HIT: health information technology
IHE: Integrating the Healthcare Enterprise
MedRec: medication reconciliation
PAR: participatory action research
