# Supplementary material for: Improving Primary Care Medication Processes by Using Shared Electronic Medication Plans in Switzerland: Lessons Learned From a Participatory Action Research Study
Source: JMIR Form Res. 2021 Jan 7;5(1):e22319. doi: 10.2196/22319 (PMC7819781; doi:10.2196/22319)
Supplement: Multimedia Appendix 1 [file formative_v5i1e22319_app1.pdf]

Plan of the meetings' goals – Summary

1st meeting: motivations and commitment (May-June)

Agenda

- Background information on the context (opportunity for questions and answers)
- Motivation and expectations with the pilot project?
- Actual use of the shared electronic medication plan?
  - => facilitate discussion on barriers and enablers to help highlight potential actions.
- Motivation to explore for the next 6 months
  - => collective commitment with defined roles.

2nd meeting: experience and refinement (September-October)

Agenda:

- Interview guide on the experience (see next page)
- Refinement of the collective commitment for the last 2-3 months

3rd meeting: synthesis and learnings (December-January)

Agenda :

- Synthesis and feedback on the initial collective commitment and motivations
- Impact of the use of the SEMP system in their practice? Efforts needed?
- Lessons learned ? Potential discussions for follow-up:
  - Potential HIT added value versus potential added value of service intervention (referent, clinical interventions,…)
  - Factors supporting regular use in practice related to the quality of the system?
  - Factors facilitating implementation in general: internal and external their organization?
  - Role of SEMP system to support collaboration between GP and pharmacy and coordination with other healthcare providers (especially hospital, homecare service) ?
  - Factors influencing the patient engagement with the SEMP ?
- What are your three main lessons learned you want to share to guide stakeholders?
- Feedbacks about the participatory action-research process together?

Interview guide : focus group on the experience (2nd meeting)

Number of patients recruited and following up? How was your experience with the patient when launching the use of a SEMP ?

Experience of the use of the SEMP

How was it to initiate the first time the SEMP?

- Roles? Tasks? Collaboration?
- Encountered Barriers? Strategies?

How do you usually use the SEMP system in your existing process ?

- How do you organize its use within your overall staff ?
- Do you associate its use with other activities/clinical intervention (e.g. medication review)?

How do you manage a change (e.g. new treatments) to ensure good communication and the accuracy of the shared information?

- Issues? Pitfalls? Risks?
- Advantage? Added-value of the SEMP?
- Formal procedure defined?

What are the added-value from the use of a SEMP?

- What are the added-value depending on some additional clinical intervention associated (e.g. medication review)

(summarize)
What can we learn from your experience using the SEMP system to better collaborate in the group?

- Anything to refine in the organization?

Reflection on the implementation

What are the factors influencing the implementation of the SEMP in your practice (apart of the design quality of the technology and integration)?

- Follow-up with barriers/enablers pointed out earlier at their organizational level and in the context (e.g. policy, financial incentives)

How the SEMP can influence the collaboration among the care professionals?

- Follow-up on some barriers or enablers pointed out earlier in the group

How the SEMP can influence the engagement of the patient?

What are the most important elements for further IT development and scaling-up the use of SEMPs for safer and more continuous medication management ?
